# Illuminating birth: exploring the impact of birthing environment lighting on labor

**DOI:** 10.3389/fgwh.2025.1599885

**Published:** 2025-07-15

**Authors:** Shenhav Albo, Orli Dahan, Omer Horovitz, David Peleg, Inbar Ben-Shachar, Yael Sciaky-Tamir

**Affiliations:** ^1^ Azrieli Faculty of Medicine, Bar-Ilan University, Safed, Israel; ^2^ Department of Multidisciplinary Studies, Tel-Hai College, Upper Galilee, Israel; ^3^ Department of Psychology, Tel-Hai College, Upper Galilee, Israel; ^4^ Department of Obstetrics and Gynecology, Ziv Medical Center, Safed, Israel

**Keywords:** natural childbirth, birth setting, perineal injuries, flow mental state, birth experience

## Abstract

**Introduction:**

Numerous factors influence the birth experience and outcomes, both positively and negatively. We aimed to investigate the relationship between the birth room environment and light condition during birth and their effects on birth method, perineal health, and birth experience.

**Method:**

A longitudinal cohort study was conducted in a medical center in Northern Israel. Participants completed self-report questionnaires during the third trimester of their pregnancy and again 72 h post-birth (T1 and T2, *n* = 126). Initially sociodemographic data and reproductive history were collected, as well as preparation and plans for birth. Data about birth outcomes and birth complications were gathered from electronic records. Perception of the birth environment and the state of consciousness during birth (T2) was assessed using a valid questionnaire that includes 36 statements indicating the state of flow.

**Results:**

Our study demonstrated a significant positive correlation between birth type and birth room light conditions. Vaginal births predominantly occured under dim light (86.36% vs. 68.3%). Moreover, a negative correlation was observed between perineal tears and dim light levels (*p* = 0.0033). Regarding maternal mental state during birth, dimmer lighting correlated with heightened experiences of Unambiguous feedback flow state (*p* = 0.003).

**Discussion:**

Dim light was correlated with higher rates of vaginal birth, fewer perineal tears, and enhanced maternal immersion during birth. Although promising, these associations are correlational and require further exploration. Our findings suggest that the birth room is not merely a physical setting but a dynamic environment where sensory cues and psychological states interact.

## Introduction

In recent years, there has been a surge in research examining the impact of the birthing room environment and design on birth outcomes and maternal experience (1–3). Ensuring a positive birth experience is vital for mothers and newborns, laying the groundwork for a healthy start. It not only encourages bonding and facilitates successful breastfeeding (4), but also impacts the mother's emotional well-being, potentially lowering the risk of postpartum depression and anxiety (5). Conversely, negative birth experiences often involve more medical interventions like instrumental births and unplanned caesarean sections, which may lead to adverse mental effects postpartum (6).

Goldkuhl et al. (3) found that the birthing room environment (i.e., physical space, human interaction, and institutional context) plays an important role in birth outcome and women's “sense of agency”. A sense of agency in childbirth refers to a woman's feeling of being in control and able to make informed decisions throughout the birthing process. It is one of the crucial factors leading to a positive birth experience (7). Balabanoff et al. (2) observed that birth room design and the type of lighting might affect melatonin production, which acts together with oxytocin to trigger birth. On the other hand, Ayerle et al. (1) could not prove that alternatively designed birthing rooms (including specific lighting types) affected the type of birth, analgesia or perineal health but found a positive effect on women's birth experience.

Women seeking a more physiologic birth sometimes opt for home births, where familiar surroundings can foster relaxation and reduce time-related stress. This environment may facilitate adherence to a natural birth process, potentially decreasing perineal injuries (8). Home births may be associated with increased maternal confidence, personalized support, and a serene environment. Greater control over the birth process, including choice of position, could contribute to improved perineal health and overall birth experience (7–9). The subjective birth experience, particularly during an unanesthetized physiologic birth, can be described by the phenomenon of flow during an intense and demanding psycho-physiological experience (10, 11). Experiencing flow means being focused on one's acts and goals, being absorbed in the moment, feeling highly confident in one's ability to succeed, and feeling accomplishment and joy during the event—even though strong pain is part of the experience (12). Usually, the extraordinary experience of flow is discussed in connection with experiences such as running a marathon (13–15) or engaging in other intense sports activities (16). A recent study demonstrate that the unique sensations and feelings of the flow mental state can successfully capture the event of childbirth, a unique, demanding psycho-physiological activity experienced by birthing women (17). Experiencing flow is perceived highly positive, psychologically and physiologically, thus considered a positive peak experience, which is empowering and might contribute to well-being (18, 19).

Aligned with the World Health Organization guidelines, healthcare providers should prioritize a positive and empowering birth experience for all women. Key recommendations include limiting episiotomies, promoting upright positions for low-risk births, and fostering a supportive environment through continuous companionship. Empowering women with choices, providing encouragement, and building confidence in their ability to give birth—are essential for a positive birth outcome (20).

The birth environment, whether at home or in a hospital, influences birth outcomes. Research suggests that hospital births result in fewer physiological births and more perineal tears (9, 21). Given that most women in industrialized societies opt for hospital births (22), it is crucial to explore whether introducing simple changes in the typical hospital birth environment could lead to better outcomes.

Given the inconclusive findings of studies on the effect of birth room design on birth outcomes, we conducted this study to investigate the potential link between the perception of birthing environment lighting and birth outcomes such as birth mode, perineal health, and maternal mental state. By examining both objective and subjective aspects of childbirth within a standard hospital setting, we sought to contribute to a deeper understanding of factors influencing birth outcomes.

## Methods

### Study setting and participants

Participants were women who gave birth at Ziv Medical Center between January 2023 and September 2023. Women were recruited during the third trimester at routine antenatal care visits, the obstetric emergency room, or the post-date clinic.

### Inclusion and exclusion criteria

Eligible participants were women aged 18–45 with a singleton pregnancy at ≥34 weeks gestation, who completed the initial T1 questionnaire before the onset of labor. Women were excluded from the analysis if they: (a) had an elective (planned) caesarean section, (b) did not complete the postpartum T2 questionnaire, (c) had missing or incomplete data on lighting perception, birth outcomes, or flow score. Only births with available data from both self-reports and medical records were included in the final analysis.

### Ethical considerations

All participants provided written informed consent before entering the study. Ethical approval was granted by the Helsinki Committee at Ziv Medical Center (approval number ZIV-0125-22).

## Measures

### Lighting conditions

Information regarding the parturient perception of birth room lighting was collected in the T2 questionnaire. Women were asked to “mark the correct statement” regarding the lighting in the birth room during most of the birth process. The options were I. The room was dark/dim II. The room was bright.

### Flow state

The 36-item Flow State Scale (12) was used to measure subjective birth experience within 72 h from birth (T2). It assessed flow as a positive peak experience—a state of complete immersion and focus, where physical and mental efforts align– on a 5-point Likert scale. The sum score ranges between 36 and 180, with higher scores indicating higher levels of experienced flow. The Cronbach's alpha reliability in the present study was 0.944.

### Birth outcomes and background variables

Information on the birth process and birth outcomes was obtained from the electronic birth records, including vaginal birth (i.e., vaginal birth with or without epidural analgesia), vacuum extraction, unplanned cesarean surgery, or planned cesarean surgery. We included information about perineal tears, including the specific tear degree. Women self-reported their ethnicity, years of education, childbirth intentions (preferred mode of birth), and childbirth preparations (courses, online forums, birth instruction books, etc.).

### Statistical analysis

For the statistical analysis, SPSS version 27 was used. A Chi-Square test investigated the relationship between birth mode and lighting conditions. The impact of lighting conditions on the severity of vaginal tears was assessed using the Kruskal–Wallis test due to the ordinal nature of the vaginal tears data and the non-normality of the distribution. Finally, the self-reported Flow State Scale scores were compared between lighting conditions using the Mann–Whitney *U* test.

## Results

**Figure 1:**
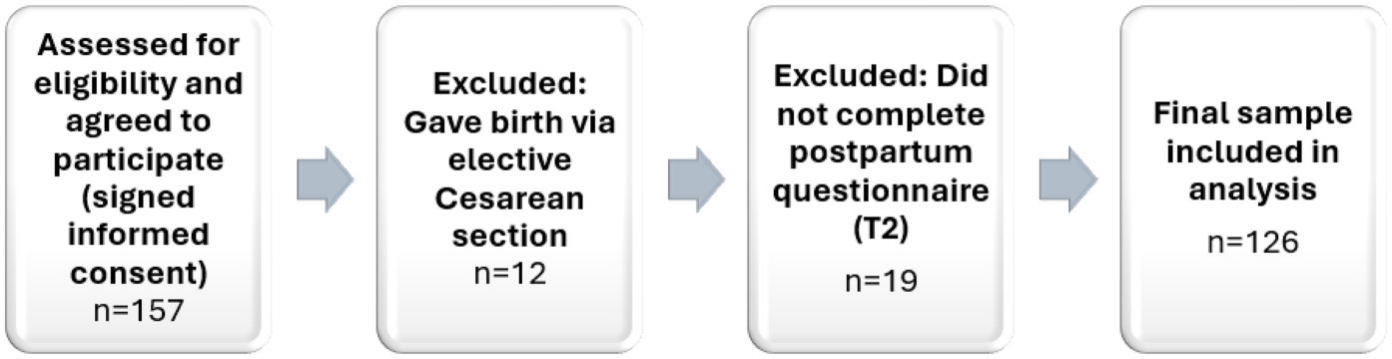
Participant recruitment and inclusion process.

In total, 157 women consented to participate. Nineteen were excluded due to incomplete T2 questionnaires, and twelve additional participants underwent planned caesarean sections. The final sample comprised 126 women who completed both questionnaires and met all eligibility criteria (see Figure 1).

The mean age of participants was 30.62 years (±4.69); the majority having an academic education (64.7%). Most participants were Jewish (70.48%). The average gravidity was 2.72 (±1.91), and the average parity was 2.32 (±1.66). A third of the study population were nulliparous (33.33%). The analysis of potential correlations between demographic variables identified no statistically significant associations. Thus, demographics were not considered possible confounders in further analyses (Table 1). In contrast, significant differences were found for two obstetrics characteristics (i.e., type of birth and vaginal tears). These variables were further examined in the statistical analysis (Table 2).

**Table 1:** Baseline demographics and clinical characteristics.

Characteristic	Bright room (*N* = 60)	Dark/Dim room (*N* = 66)	*P* value
Age _(y)_	30.5 ± 3.9 (23–37)	30.7 ± 5.4 (21–42)	*p* = 0.09^a^
Educational Level			*p* = 0.29^b^
Non-Academic	26 (43.3)	18 (27.3)	
Academic	35 (56.7)	48 (72.7)	
Gravidity	2.6 ± 1.7 (1–6)	2.7 ± 2.1 (1–8)	*p* = 0.07^a^
Parity	2.2 ± 1.5 (1–6)	2.4 ± 1.8 (1–8)	*p* = 0.08^a^
Nulliparity	20 (33.33)	22 (33.33)	*p* = 0.16^b^

Values are presented as *n* (%) or mean ± SD (range).

^a^ Mann–Whitney Test.

^b^ Chi-square Test.

**Table 2:** Labor parameters and interventions.

Characteristic	Bright room (*N* = 60)	Dark/Dim room (*N* = 66)	*P* value
Analgesia			0.23^b^
Without	12 (20.0)	20 (30.3)	
N_2_O^a^	2 (3.3)	3 (4.5)	
Epidural	40 (66.7)	42 (63.7)	
Spinal	3 (5.0)	1 (1.5)	
General anesthesia	3 (5.0)	-	
IOL^b^			0.64^b^
No induction	31 (51.7)	37 (56.0)	
Induction of labor	29 (48.3)	29 (44.0)	
Type of birth			0.04^b^
Vaginal birth	41 (68.3)	57 (86.36)	
Vacuum	13 (21.7)	2 (3.33)	
Unplanned Cesarean section	6 (10)	7 (11.67)	
Birth weight (g)	3,392.2 ± 375.7	3,473.5 ± 375.3	0.99^a^
Episiotomy	11 (18.33)	10 (15.15)	0.63^b^
Vaginal tear			0.01^b^
0	24 (46.0)	43 (57.0)	
1–2	34 (50.0)	23 (43.0)	
3–4	2 (4.0)	0 (0.0)	
Flow^d^	104.9 ± 29.2	116.2 ± 25.0	0.03^c^

N_2_O, Nitros Oxide; IOL, induction of labor.

Values are presented as *n* (%) or mean ± SD (range).

^a^ Mann–Whitney Test.

^b^ Chi-square Test.

^c^ Student Independent *T*-test.

^d^ Flow measured with the Flow state scale (FSS), Jackson & Marsh, 1996.

Our study identified a significant association between birth mode and light perception (*p* = 0.033). *post-hoc* comparisons showed that vaginal births predominantly occurred under dark/dim lighting (86.36%, *p* = 0.001), while assisted vaginal (vacuum) births were more frequent in bright room conditions. Specifically, 21.7% of women delivering in bright rooms had vacuum deliveries compared to only 3.33% of those giving birth in dim lit rooms (*p* = 0.031). Unplanned Cesarean section rates did not differ between the rooms (Table 2).

In our cohort, there were no 4th-degree tears. The analysis revealed a statistically significant difference in the degree of vaginal tears under the different lighting conditions during birth (dim or bright, *p* = 0.003). *post-hoc* comparisons showed that women who gave birth in dark/dim lighting predominantly did not have vaginal tears (57.0%, *p* = 0.006), while those giving birth in bright room conditions predominantly had 1st and 2nd tears degree (50.0%, *p* = 0.003). No differences were found for 3rd tears degree (Table 2).

Evaluating differences in self-reported flow during childbirth based on lighting conditions in the birth room showed that women in dimly lit rooms reported higher flow levels (*p* = 0.033). Simply, women in dimly lit rooms experienced greater flow (116.18 ± 25.02) than those in well-lit rooms (104.93 ± 29.23, *p* = 0.029—Table 2).

## Discussion

Our study combined self-reported experiences and data collected from clinical records to explore the relationship between birth environment, birth outcomes, and maternal states of consciousness. We found a high correlation coefficient between parturient 's report of dim light in the birth room and an increased likelihood of a successful vaginal birth, fewer perineal tears, fewer episiotomies, and a heightened tendency to experience the flow mental state.

### The association between a dimly lit room and vaginal birth

Given the impact of the birth method on postpartum mental health, it's necessary to explore avenues that promote physiological births (23). Recent research highlights the impact of dimmed lights in birth rooms as directly associated with fewer emergency medical interventions (24). Our research demonstrated a significant positive association between vaginal birth and dim light in the birth room. As caesarean section rates rise globally, there is growing concern about the health implications for both mothers and infants (1). Optimal birth environments are characterized by dim light, minimal noise, limited medical intervention, and professional continued support. These factors lead to low stress, privacy, safety and calmness (2). Dimly lighted birth rooms contribute to privacy; empowering birthing women by fostering feelings of control and autonomy. This intimate environment promotes maternal confidence in her ability to have a natural birth (8). From a hormonal perspective, the relationship between circadian rhythm and birth time has been called “the biorhythm of birth,” suggesting that myometrial activity is maximal at night, when melatonin levels are higher (25). Previous studies suggest that melatonin may act in synergism with oxytocin on myometrial receptors to enhance uterine contractility (26, 27). Moreover, it has been suggested that low, dim light may stimulate oxytocin production and influence melatonin levels, further contributing to the hormonal conditions favorable for labor progression (2). While this pathway is biologically plausible, it was not directly examined in our study and remains hypothetical in our context.

Creating a birth environment reminiscent of a private home with dim lighting helps birthing women focus, feel comfortable, and experience fewer disruptions (8). By promoting such conditions, it may be possible to support higher rates of vaginal birth and reduce the likelihood of unplanned caesarean sections.

### A link between a dimly lit room and perineal tears

Perineal trauma affects 53%–79% of women after vaginal birth (28, 29). First and second-degree lacerations are common, but severe tears (3rd and 4th degree) are more prevalent in nulliparous women (29). Risk factors include maternal position, operative birth, epidural analgesia (21, 30), ethnicity, nulliparity, maternal age, fetal weight, perineal edema, birth stage, and hospital birth (9, 21, 29). Lithotomy position, common in hospitals, increases risk (30). Episiotomy, once routine, is now less common due to a lack of proven benefits. Its prevalence is 12% in the US (31) and 14.3% in Israel in 2022. The rate of 3rd and 4th-degree lacerations that year was 0.57%, while at the study site the rate was 10% and 0.95%, respectively (Data presented by the Israeli Society of Maternal-Fetal Medicine—March 2024-personal communication).

Perineal injuries and anal sphincter tears, common complications of vaginal birth, can result in pain, discomfort, incontinence, and long-term psychological distress. Approximately one-third of women report a traumatic childbirth experience (6). Tears are less common among women giving birth at home when compared to those giving birth in the hospital (8, 9) and the prevalence of perineal tears and trauma has increased in correlation with an increase in hospital births (8).

Various perineal management strategies, including massage, support, warm compresses, positioning, and delayed pushing, have been implemented to prevent perineal trauma during and before childbirth (31). This study investigated the potential of a darkened birthing environment to reduce perineal trauma, perhaps by promoting muscle relaxation.

Although the connection between a dark birth room and less severe perineal tears during childbirth is a topic with limited research, there are some potential explanations for possible existing correlation. A relaxed birthing environment might allow for more natural positioning and activation of birthing reflexes, potentially reducing the need for interventions that could contribute to vaginal or perineal tears (32).

Fear and pain are integral parts of birth. Melatonin has been proposed to influence pain regulation and may reduce the need for pharmacological analgesia during birth (33). In a qualitative study, midwives noted a correlation between feelings of fear during childbirth and the occurrence of tears, suggesting that maintaining maternal sense of security, autonomy and feeling in control of the birthing process, may help reduce the likelihood of such complications (8, 9, 34). Women also want to avoid pain by pushing as much as they can. This study suggests that a dimmed birth room scenery is similar to a private room or home. It helps create an intimate environment inside the hospital. It thus improves the likelihood of achieving an ideal outcome for the birthing woman, both emotionally and physically, characterized by a reduced frequency of perineal trauma (8). During a spontaneous birth process, the hormones that start and maintain birth also sustain the instinctive emotions and behavior of the birthing women (10). The biochemical processes of normal birth promote pain reduction as birth progresses (10, 11). The simultaneous increase of brain levels of oxytocin, which act synergistically with melatonin (for initiation of contraction), prolactin, and endorphins, modify women's pain experience during physiological birth and enables the birthing woman to focus and retreat (11).

### A link between a dimly lit room and the flow mental state

The application of flow theory to childbirth warrants clarification. While flow is traditionally studied in the context of goal-directed skilled activities such as sports or music (12), its core phenomenological features—intense focus, altered time perception, loss of self-consciousness, and deep embodiment—can also emerge in internally guided processes like physiological birth (4, 5, 10, 11). Women often describe labor as involving strong bodily cues, diminished awareness of external stimuli, and immersion in the rhythm of contractions (10, 11). These experiential elements parallel to key components of the flow state (12), even when volitional control is limited. Thus, childbirth may evoke a unique form of embodied flow worthy of empirical exploration.

Other studies have identified associations between the flow state and physiological birth (17). Our study found a similar association between experiencing the flow state and giving birth in a dimly lit room. This may stem from a stronger mind-body connection facilitated by the darker environment. During childbirth, the body provides natural feedback through contractions and birthing urges. In a dark environment, the birthing person might rely more on internal cues and sensations to guide their actions, further strengthening the mind-body connection. One proposed explanation from a neurofunctional perspective is the transient hypofrontality mechanism, which is the reduced activity in the frontal cortex that correlates with sensations of calm, less pain, less anxiety, and being in inner focus (13, 14). This theoretical model suggests that reduced frontal cortical activity may correlate with sensations of calm, reduced pain, and inward focus (35).

In relation to the darker environment, the transient hypofrontality mechanism, and the experience of flow, additional contributing factors may include reduced external distractions and enhanced feelings of safety. Darkness minimizes visual stimuli; thus, in the natural birth process context, it can also reduce distractions (11, 23, 35). Perhaps allowing the birthing woman to focus inward on their body's sensations and natural birthing urges. This can lead to heightened awareness and connection with their body.

As discussed before, a dark environment might promote melatonin production, potentially working with oxytocin to enhance its effects. Oxytocin is crucial for contractions but can also contribute to a feeling of focus (10, 11, 35). Moreover, the feeling of safety and privacy associated with a dark environment can promote relaxation and reduce stress hormones (35, 36). In the context of the flow sensation, it is reasonable that a calmer state of mind may allow for a better connection with one's bodily sensations during the challenging birth process.

While our study did not directly assess hormonal or neural activity, previous research has linked dim lighting with increased melatonin production and hypothesized synergy with oxytocin in facilitating labor. In our study, these pathways remain theoretical and were not empirically tested. Similarly, the proposed link between dim environments and the transient hypofrontality mechanism—associated with inward focus and pain modulation—was not evaluated. Future studies incorporating hormonal assays or neuroimaging could help clarify these mechanisms.

## Strengths, limitations, and directions for future research

Like all observational studies, our findings must be interpreted with caution due to several methodological limitations, including issues related to sampling, measurement, and generalizability, as discussed below. Nonetheless, the study also has notable strengths: it integrated subjective and clinical data, employed a validated flow scale with high internal consistency, and addressed an underexplored yet modifiable environmental factor—birth room lighting.

A key limitation of this study concerns the absence of objective contextual and environmental measures. For example, lighting conditions were assessed solely through subjective maternal reports and not corroborated by external observations or digital sensors (e.g., lux meters). Similarly, a range of contextual birth-related variables—such as birth position, primary caregiver (e.g., midwife vs. obstetrician), use of analgesia, and labor interventions—were not systematically measured or controlled for. These unmeasured variables may have contributed to both birth outcomes and flow experiences, thereby confounding the observed associations. Future research would benefit from more comprehensive, multi-source data collection to better account for the complex interplay between environment, provider behavior, and maternal experience.

In addition, the measurement of the flow state relied entirely on self-reported retrospective evaluations. Although the Flow State Scale is a validated tool and was administered within 72 h postpartum, it remains sensitive to outcome-dependent reporting bias. Women who experienced less physically or emotionally demanding births may have been more inclined to interpret their experience as immersive, harmonious, or optimal. This poses a potential confounding bias, as positive birth outcomes may influence how women retrospectively evaluate their cognitive-emotional state during labor. Future studies could improve reliability by incorporating real-time assessments or complementary observational data.

Beyond methodological limitations, childbirth itself involves an intricate interplay between neurohormonal, physiological, cognitive, and emotional factors. This system exhibits a sensitive feedback loop (37), making it difficult to isolate the causal influence of any single variable. In light of this complexity, exploring correlations and interactions—rather than seeking singular cause-and-effect explanations—may offer more ecologically valid insights.

Some of the subgroup analyses conducted in this study involved relatively small cell sizes, particularly in cases of vacuum-assisted deliveries and third-degree perineal tears. As a result, these comparisons may be underpowered and carry a greater risk of both Type I and Type II statistical errors. These findings should therefore be interpreted with caution, and future research with larger sample sizes is recommended to confirm these associations.

Finally, the generalizability of the findings is constrained by the sample composition. The study was conducted in a single hospital in northern Israel with a relatively homogenous population—primarily academically educated, Jewish women. It is possible that women with higher education or cultural familiarity with research were more likely to consent to participation. These demographic and cultural characteristics may limit the applicability of results to more diverse or multinational populations. Replication in varied clinical and cultural contexts is needed to establish the broader relevance of the observed associations.

## Conclusion

In this cohort observational study, we identified associations between birthing room lighting, mode of birth, perineal tears, and the mental state of women during childbirth. Dimmer lighting was linked to higher rates of vaginal birth, fewer perineal tears, and a greater likelihood of experiencing a flow mental state.

While preliminary, these results support the notion that the birthing room environment — particularly lighting — may subtly influence both physiological outcomes and mental states during labor. Clarifying the mechanisms behind these associations requires further multidisciplinary and experimental research.

By examining environmental factors such as light alongside neurohormonal and psychological processes, we may gain deeper insight into how to optimize childbirth experiences and outcomes. Importantly, these associations remain correlational, and causal links must be explored in future studies.

## Data Availability

The datasets presented in this article are not readily available because the data sets are to be used by the research group members. Personal info was annonimized before shared with the statistician for evaluation. The identifiers of the participants is saved on a secure file in our hospital server. We did not receive agreement from patients to share this data. Requests to access the datasets should be directed to yael.st@ziv.gov.il
